# Intelligence

**Published:** 1830-08

**Authors:** 


### INTELLIGENCE.

### MEDICAL ESTABLISHMENT OF THE COURT.

We understand that the following appointments have been made:

*Physicians in ordinary to his Majesty:* Sir Gilbert Blane, Bart., Sir Henry Halford, Bart., and Sir M. Tierney, Bart.

*Physicians extraordinary:* Sir James Macgrigor, Dr. Maton, Dr. Warren, and Dr. Macmichael.

*Physician to the Household:* Dr. Francis Hawkins.

*Sergeant Surgeons:* Sir E. Home, Bart. and Sir A. Cooper, Bart.

*Surgeons extraordinary:* R. Keate, Esq., B. C. Brodie, Esq., H. Earle, Esq., and J. M. Arnott, Esq.

*Physicians in ordinary to the Queen:* Sir H. Halford, Bart., and Dr. C. M. Clarke.

*Physicians extraordinary:* Dr. Turner, Dr. Southey, and Dr. Locock.

*Surgeon to the Queen and to her Majesty's Household:* Robert Keate, Esq.

His Royal Highness the Duke of Sussex has appointed Dr. Seymour, physician to St George's Hospital, to be one of his physicians in ordinary.

---

### OFFICIAL ACCOUNT *of the* POST-MORTEM EXAMINATION *of* HIS LATE MAJESTY.

The body exhibited but little sign of putrefaction; and the anasarca had disappeared, excepting some slight remains of it in the thighs.

Notwithstanding the apparent emaciation of his Majesty's person, a very large quantity of fat was found between the skin and the abdominal muscles.

*Abdomen.* The omentum, and all those parts in which fat is usually deposited, were excessively loaded with it. The abdomen did not contain more than an ounce of water.

The stomach and intestines were somewhat contracted; they were of a darker colour than natural, in consequence of their containing mucus tinged with blood; and in the stomach was found a clot of pure blood, weighing about six ounces.

The liver was pale, and had an unhealthy granulated appearance.

The spleen, although larger than usual, was not otherwise diseased, and the pancreas was in a sound state.

The sigmoid flexure of the large intestine (the colon) had formed unnatural adhesions to the bladder, accompanied by a solid inflammatory deposit, of the size of an orange.

Upon a careful examination of this tumor, a sac or cavity was found in its centre, which contained an urinary calculus, of the size of a filbert, and this cavity communicated, by means of a small aperture, with the interior of the bladder at its fundus. In other respects the bladder was healthy, and the prostate gland did not appear to be enlarged. The kidneys were also free from disease.

*Thorax.* Two pints of water were found in the cavity of the right side, and three pints and three quarters in the left side of the chest. The left lung was considerably diminished.

The lower edge of each lobe of the lungs had a remarkable fringe, which, upon examination, was found to be formed by a deposit of fat.

The substance of the lungs had undergone no change of structure, but the mucous membrane lining the air-tubes was of a dark colour, in consequence of its vessels being turgid with blood.

The pericardium (or heart-purse) contained about half an ounce of fluid; but its opposite surfaces in several parts adhered to each other, from inflammation at some remote period.

Upon the surface of the heart and pericardium there was a large quantity of fat, and the muscular substance of the heart was so tender as to be lacerated by the slightest force: it was much larger than natural. Its cavities upon the right side presented no unusual appearance, but those on the left side were much dilated, more especially the auricle.

The three semilunar valves at the beginning of the great artery (the aorta) were ossified throughout their substance, and the inner coat of that blood-vessel presented an irregular surface, and was in many parts ossified.

The original disease of his Majesty consisted in the ossification of the valves of the aorta, which must have existed for many years, and which, by impeding the passage of the current of blood flowing from the heart to the other parts of the body, occasioned effusion of water into the cavities of the chest and in other situations. The mechanical impediment to the circulation of the blood also sufficiently explains those other changes in the condition of the body which were connected with his Majesty's last illness, as well as all the symptoms under which the King had laboured.

The immediate cause of his Majesty's dissolution was the rupture of a blood-vessel in the stomach.

HENRY HALFORD.
MATTHEW JOHN TIERNEY.
ASTLEY PASTON COOPER.
C. B. BRODIE.

---

### OBSTETRIC SOCIETY.

*To the Editor of the London Medical and Physical Journal.*

SIR: Having been directed to furnish you with a short account of the Obstetric Society, I send you the inclosed.

I am, Sir, your obedient servant,

F. H. RAMSBOTHAM, *Hon. Sec.*

14, *New Broad street; June* 1, 1830.

In consequence of the numerous instances which had occurred within the knowledge of many medical practitioners, of great mischief produced by the indiscriminate practice of Midwifery, some gentlemen were induced to form themselves into a Society at the end of the year 1825, for the purpose of promoting obstetrical knowledge, and of protecting the public from the injuries to which they were exposed. The Obstetric Society originally consisted of thirty-one members, and since its establishment no others have been added: it includes the names of most of the present and former physicians and surgeons of the obstetrical institutions, as well as most of the lecturers on midwifery in this metropolis. A subscription of two guineas from each member has been found sufficient to meet all the expence which it has up to the present time incurred.

As it was the great object of this Society to act in unison with the three medical corporate bodies; and as not one of those bodies acknowledged midwifery as a part either of Physic or Surgery, by examining candidates on their capability to practise this branch of the profession, the most proper and direct course for the Society to pursue was thought to be that of addressing the three corporate bodies, stating to them strongly and broadly the feelings which the Society entertained on the subject, and requesting to know whether they were disposed to obviate the causes of the calamities complained of. Provided, however, the constituted medical authorities did not consider themselves invested with sufficient powers, or possessing those powers did not feel inclined to exercise them for the public good, the Society declared their intention of appealing to the legislature for the accomplishment of that object, which would then appear to be attainable by no other means. They suggested at the same time that all the desired advantage might be gained, and the proposed protection of the community would be most easily secured, by each body inquiring for itself, through its own examining board, into the extent of the knowledge of midwifery possessed by each candidate who came before them. This mode of proceeding seemed to the Society to offer the fewest obstacles and be most free from objections, as it would leave the relative situations of the three corporate bodies undisturbed, whilst it would secure to the public the certainty of having qualified practitioners in midwifery, as well as in the other departments of the profession.

To this letter, which was dated March 20th, 1826, the apothecaries first replied, and in their answer (bearing date April 4,) they stated that they were well satisfied of the existence of abuses occasioned by the indiscriminate practice of midwifery, and would be happy, if it were in their power, to render assistance in promoting the laudable object of the Society; but they were of opinion that their Act did not give them any authority to superintend and regulate the practice of midwifery; they considered the subject well entitled to parliamentary interference, and stated their willingness to conduct examinations in midwifery, provided parliament would commit to them the responsibility. In the reply of the College of Physicians, on June 9th, the grand question was completely evaded: it merely consisted of a resolution to the effect that "the College was best satisfied when midwifery was practised by those who had been found, on examination before them, to be competent to the exercise of the profession at large by their knowledge and acquirements; that the delivery of women was an art of manual skill, and therefore in the province of surgery; and that the treatment of the diseases of pregnancy and the puerperal state was a part of the general practice of physic, and as such liable to the inquiry of the Censor's Board;" and, in the answer of the College of Surgeons, which was not received till July the 19th, four months after the Society's address was presented, the positions laid down by the Society were tacitly admitted as true, by the expressions "that the College were not invested with sufficient power to obviate the evils stated by the Society, to arise from the want of legislative regulations over the practice of midwifery."

The Society now felt that its next endeavour might with propriety be directed towards obtaining the interference and powerful aid of government in a cause which so intimately concerned the whole community, and a resolution to the effect of addressing his Majesty's secretary of state for the home department was passed in January 1827. Still, however, being willing to afford to the three corporate bodies another opportunity of co-operating with the Society in the settlement of the question, the Society resolved to address them once more, apprising them, at the same time, of their determination to apply for the assistance of government.

The replies of the chartered bodies on that occasion followed each other more speedily; first from the surgeons on February 17th, 1827, who stated that their College was instituted solely for the purpose of examining and attesting the capability of persons to practise the art and science of surgery; consequently, being solicitous that those persons who conduct the examinations should be particularly skilled in surgery, the College had excluded from the court of examiners those surgeons whose time and attention had been occupied in learning and practising the obstetric and pharmaceutical departments of medicine; that the council of their College did not perceive how it could compel even its own members to submit to examination in midwifery; that no tribunal for examining and attesting the capability of persons to practise midwifery had yet been instituted; but that the College would willingly form a board of examination for the midwifery department of medicine, provided the government of the country deemed it necessary, and would give them the requisite authority for that purpose.

On February 22 the apothecaries returned an answer, referring the Society to their former letter; and, on February 27th, the physicians replied, who declined giving any more explicit answer.

The Society then addressed a memorial to Mr. Peel, March 20th, 1827, in which the replies from the three corporate bodies were commented upon, the great responsibility which attaches to the practitioner in midwifery demonstrated, the necessity that exists for every medical man making himself acquainted with that branch of the profession, and the impossibility of detaching it from the general science of physic and surgery, or of studying it separately, clearly shown. It was also brought before the notice of the secretary of state, that instruction on midwifery and the diseases of women and children, at that time, constituted no necessary part of the courses of lectures, as required for examination on medicine and surgery; and the object of the memorial was declared to be to oblige all who present themselves for examination before the three corporate bodies, to procure such information on the subject of midwifery as should give them competency to practise it; and to induce the examiners not to neglect the inquiry into such competency in those who present themselves before them as candidates for admission in their respective bodies. It was urged that the protection sought might be best afforded by the by-laws of the corporate bodies being so altered as to ensure professional instruction and examination in this branch of medical science, and by the repeal of those other by-laws, or the discontinuance of those customs by which men who have added practical information on midwifery to their other acquirements are excluded from the examining Boards. It suggested that the same power which was given by Act of Parliament to the corporate bodies to prevent unqualified persons practising the other branches of medicine and surgery, should be extended also to midwifery; and, lastly, it declared that the members of the Society "could not allow themselves to be selected for the purpose of forming a *separate Board* for the regulatiou of the practice of midwifery, as such a proceeding would imply a *partial* acquaintance with a profession which they had studied as a *whole*, and which they had practised as a *whole*, in common with the other members of the bodies to which they individually belong."

At the same time a communication was made to Mr. Peel, that a deputation of the Society had been empowered to wait on him, for the purpose of elucidating whatever might appear imperfectly explained in the memorial, and the right honourable Secretary appointed that those gentlemen should attend him on the fourth day after the receipt of the papers. At this interview, Mr. Peel stated he had read over attentively the memorial and correspondence; he approved the intention of the Society; admitted the importance of its objects, and promised to refer the memorial to the three corporate bodies, whose subsequent answers to the Secretary of State were transmitted to the Society in July 1827.

In their official answer, the College of Physicians considered the examinations entered into at the College on diseases of women and children a sufficient test of the candidate's knowledge on those points, and the act of delivery an operation of a surgical nature. Yet the surgeons, in their reply, far from agreeing with the latter position, contended that, by admitting practitioners in midwifery into their council, they would weaken that respect the public now entertains for, and the confidence it now has in, that council; they observed "they had hitherto elected as examiners such surgeons as they believed had, in the early period of their lives, been accustomed to pass their days in hospitals, and their nights in the study of their profession, and not in the avocation of a lying-in chamber. They, however, informed the Secretary of State that they had, since the Society's first application to them, passed a resolution, requiring certificates of attendance on two courses of lectures on midwifery from each candidate for a surgical diploma.

The Society of Apothecaries, on the other hand, in their reply, considered the object of the Obstetrical Society as tending highly to the benefit, welfare, and happiness of the community, and to the usefulness of the medical profession." They "called the serious attention of the Secretary of State to the subject, and expressed a hope that some legislative measure might be devised which would in future afford a security to the public that practitioners in midwifery were well educated in that branch of the medical profession, and fully competent to the practice of it." But, although perfectly convinced of the propriety and necessity of instituting examinations on this subject, they thought the powers they possessed did not authorise them to undertake such a duty.

The statements brought forward, and arguments used, by the three corporate bodies on this occasion, demanded severally the notice of the Society; and accordingly a second memorial was laid before Mr. Peel, bearing date March 21, 1828, in which the Society, after disposing of those replies, submitted to his consideration an easy and simple plan for placing the practice of midwifery on a respectable footing, viz. that the colleges of physicians and surgeons should be recommended to annul their respective by-laws, by which the Fellows of the former and the members of the Court of Examiners and Council of the latter College, are precluded from the practice of midwifery; and that they should also be recommended to examine all their candidates on midwifery, as well as on the other branches of medicine and surgery. While, to the Socicty of Apothecaries, also, authority should be given to institute examinations on midwifery.

In the month of January 1829, the committee of the Society was informed by one of its members, that he had had an interview with Mr. Peel on the subject of the Society's second memorial, in which Mr. Peel entered on the different points suggested to him with the greatest kindness and most deliberate attention: he promised to refer that part of the memorial containing the Society's propositions to the corporate bodies for their reconsideration; to see the leading members of the Physicians and Surgeons' Colleges on the subject; and, having so done, to forward the replies from the medical bodies to the Society.

After the lapse of five months, during which time no fresh communication reached the Society, the chairman having written to Mr. Peel, requesting to know whether any reply had been forwarded to him from the Colleges of Physicians or Surgeons, received from that gentlemen a note, inclosing a paper which had been delivered to him a few days before by the president of the College of Physicians. In this paper the question was approached for the first time with something like earnestness by the College of Physicians, the president, having briefly adverted to the history, constitution, and objects of the College, said "they could not repeal their by-laws respecting practitioners in midwifery; but proposed, in the name of the College, that, as often as any physician offered himself for examination preparatory to his licence to practise and declared his intention of adding midwifery to the practice of physic, he should be examined before the Censor's Board, by some licensed physician at that time practising, or who has practised midwifery, who should be called in as an assessor for the purpose of inquiring into his qualifications in the manual branch of that art." No reply has up to this time been received by the Society from the College of Surgeons. The Company of Apothecaries, however, in accordance with their declaration made both to the Obstetric Society and Mr. Peel, have evinced a strong desire to forward the views of the Society: the chairman of the examining Board of that body called upon the secretary of the Society in August last, to inquire how far the Society had obtained their objects in regard to the Colleges of Physicians and Surgeons, and in what way the Apothecaries' Company could further assist the Society. He said they had resolved to require certificates of attendance on two courses of lectures on midwifery and the diseases of women and children from each candidate. They were still of opinion that their Act did not give them the power to examine on midwifery; they would be willing to undertake any duties of examination in midwifery that the legislature might think proper to require of them, but they were all agreed that any such extension of their Act must come from the government, on the suggestion of the Obstetric Society, and was not to be considered as solicited and promoted by the Apothecaries' Company.

In consequence of this spontaneous declaration on the part of the Apothecaries' Company, the committee of the Obstetric Society addressed Mr. Peel once more, calling his attention again to the objects and wishes of the Society, and to the determination of the corporate bodies; and ventured to request the Secretary of State to obtain for them the opinion of the law officers of the Crown as to the power possessed by the College of Surgeons and Apothecaries' Company with regard to examinations in midwifery; in order that, if this opinion should be favorable, the two bodies first mentioned might at once act upon it; but, should it prove unfavorable, the Society then hoped the right honourable Secretary would concede to these two bodies the requisite powers for that purpose, and at the same time sanction the introduction into the House of Commons of a Bill rendering it penal for any man to practise midwifery who is not a legally authorised physician, surgeon, or apothecary.

This letter called forth from Mr. Peel, in Dec. 1829, an answer to the effect that, if the Apothecaries' Company were uncertain as to the full extent of the powers which they legally possess, and were desirous of obtaining a legal opinion on the point, he must leave it with the Board to exercise their discretion in respect to taking such an opinion. He must decline making a special reference from the Home Department to the law officers of the Crown upon the subject; neither could he undertake the introduction into the House of Commons of a Bill such as was proposed by the Society.

An abstract from Mr. Peel's letter was forwarded to the Company of Apothecaries, and a reply from the secretary to the Court of Examiners was received by the Society in the beginning of February of this year, inclosing the opinion of the attorney and solicitor general that the Court of Examiners of the Apothecaries' Company have no power by the Act of Parliament to examine in the art of midwifery, which is in no respect comprised in the Act.

At the next meeting of the committee, it was resolved that the chairman of the Society should wait upon Mr. Peel as a deputation from the Society, for the purpose of forwarding the objects in view. This resolution was complied with: the conversation was principally directed towards the offers made by the Colleges of Physicians and Surgeons; and the opinion of the attorney and solicitor general respecting the power possessed by the Society of Apothecaries under their Act was brought forward.

The Society was next called together on the 18th of this month, and it was determined that the three corporate bodies should be again addressed. It was suggested to the College of Physicians, that the regulation they proposed should be extended to all candidates who present themselves for a licence at the Censor's Board, instead of being limited to those only who, at the time of their examination, might declare their intention to practise midwifery: for it appeared to the Society it might happen that candidates who had no intention to practise midwifery when they applied for a licence, might be inclined, at a future period, to add that branch of practice to the practice of physic; and it seemed but just that all physicians who were not precluded by the established rules of the College from practising midwifery should prove before competent judges their fitness for that duty.

To the College of Surgeons the Society expressed their satisfaction at the regulations they have adopted requiring from their candidates' certificates of attendance upon lectures on midwifery, and trusted that they would see the necessity of instituting examinations by their own examining Board on this branch of surgical science: and to the Society of Apothecaries they offered their thanks for the attention which their former letters had received from that Society, and expressed their desire to render their assistance to them in endeavouring to obtain an Act of Parliament, authorising the Society of Apothecaries to examine those candidates who came before them as to their qualification to practise midwifery. The Society now wait for any other communication from the three corporate bodies.

It must be observed that, on the institution of the Obstetric Society, it was resolved that its objects should embrace the regulation of the practice of midwifery with regard to female as well as male practitioners; but it has been thought proper to direct the attention of his Majesty's government at present only to those persons practising midwifery, over whom the medical bodies ought to possess some control : no mention has, therefore, been made, as yet, of the injuries to which the poorer classes of the community are exposed, from the mismanagement of women imperfectly educated in the duties of their profession.

The Society, however, has not been unobservant of such dangers, and is most desirous that some means may hereafter be adopted to correct this grievous abuse.

It is also to be remarked, that the Society was originally formed with the sole intention of ensuring the welfare of the public, as far as midwifery is concerned, and that at one of their first meetings it was resolved that, when the object of regulating the practice of midwifery shall have been carried into effect, its existence shall become extinct.

---

### MONTHLY LIST OF MEDICAL BOOKS.

### METEOROLOGICAL JOURNAL,

By Messrs. Harris and Co. Mathematical Instrument Makers, 50, High Holborn.

| June | Moon. | Rain gauge. | Thermom. 9 a.m. | Thermom. max. | Thermom. min. | Barometer. 9 A.M. | Barometer. 10P.M. | De Luc's Hygrom. 9A.M. | De Luc's Hygrom. 10P.M. | Winds. 9 A.M. | Winds. 10P.M. | Atmospheric Variations. 9 a.m. | Atmospheric Variations. 2 p.m. | Atmospheric Variations. 10 p.m. |
|---|---|---|---|---|---|---|---|---|---|---|---|---|---|---|
| 20 | ● | | 60 | 68 | 46 | 29.46 | 29.43 | 50 | 52 | WNW | WNW | Fine | Fine | Fine |
| 21 | | | 52 | 57 | 48 | .37 | .32 | 54 | 55 | WNW | ENE | —— | —— | Rain |
| 22 | | .29 | 53 | 63 | 43 | .24 | .53 | 55 | 53 | NNE | N | Rain | Fine | Cloudy |
| 23 | | | 55 | 63 | 54 | .71 | .73 | 53 | 51 | N | NW | Fine | Cloudy | Fine |
| 24 | | | 63 | 65 | 56 | .74 | .73 | 50 | 57 | NNW | ENE | —— | Fine | —— |
| 25 | | .31 | 60 | 64 | 60 | .63 | .51 | 62 | 61 | ENE | ENE | Rain | —— | Cloudy |
| 26 | | | 65 | 72 | 58 | .56 | .65 | 58 | 54 | SSW | SSW | Cloudy | —— | Fine |
| 27 | | | 67 | 71 | 57 | .68 | .72 | 52 | 52 | NW | W | Fine | —— | —— |
| 28 | ☽ | | 69 | 74 | 60 | .72 | .66 | 51 | 51 | S | SSW | —— | —— | Cloudy |
| 29 | | | 68 | 71 | 58 | .71 | .83 | 50 | 49 | WSW | W | —— | —— | Fine |
| 30 | | | 63 | 69 | 55 | .87 | .89 | 48 | 51 | W | S | —— | —— | —— |
| July 1 | | | 65 | 68 | 58 | .78 | .62 | 51 | 54 | ESE | ESE | —— | —— | Cloudy |
| 2 | | .40 | 62 | 69 | 56 | .55 | .44 | 56 | 56 | SE | S | Rain | Show'ry | Cloudy |
| 3 | | | 62 | 67 | 55 | .35 | .52 | 54 | 54 | ENE | NW | Show'ry | —— | Show'ry |
| 4 | | .30 | 60 | 69 | 57 | .64 | .76 | 52 | 52 | SW | SW | Fine | Fine | Fine |
| 5 | | | 63 | 67 | 59 | .84 | .88 | 55 | 54 | WNW | WNW | Cloudy | Cloudy | Fine |
| 6 | ○ | | 67 | 67 | 60 | .84 | .68 | 54 | 56 | WSW | WSW | Fine | Fine | Show'ry |
| 7 | | .06 | 65 | 67 | 56 | .47 | .50 | 53 | 53 | SW | WSW | Rain | Fine | Fine |
| 8 | | | 63 | 65 | 52 | .55 | .42 | 52 | 51 | W | WSW | Fine | —— | Rain |
| 9 | | | 59 | 63 | 55 | .30 | .42 | 51 | 50 | W | W | Cloudy | —— | Fine |
| 10 | | | 61 | 65 | 53 | .61 | .72 | 49 | 49 | W | W | Cloudy | —— | —— |
| 11 | | | 59 | 65 | 55 | .64 | .48 | 53 | 55 | SW | SW | Fine | Cloudy | —— |
| 12 | | | 63 | 66 | 53 | .40 | .76 | 58 | 53 | SSW | W | Cloudy | Fine | —— |
| 13 | ☾ | | 61 | 70 | 56 | .96 | 30.00 | 51 | 50 | WSW | WSW | Fine | —— | —— |
| 14 | | | 64 | 72 | 61 | .95 | 29.86 | 50 | 49 | SW | SW | —— | —— | —— |
| 15 | | | 68 | 70 | 60 | .81 | .82 | 49 | 51 | SSW | SSW | —— | —— | —— |
| 16 | | | 66 | 66 | 55 | .85 | .92 | 51 | 50 | SW | SW | —— | —— | —— |
| 17 | | | 65 | 67 | 54 | .87 | .74 | 49 | 55 | SW | SSW | Cloudy | Cloudy | Rain |
| 18 | | | 59 | 64 | 55 | .66 | .70 | 58 | 63 | SW | SW | Rain | Rain | —— |
| 19 | ● | .66 | 64 | 65 | 57 | .81 | .91 | 62 | 59 | WSW | SW | Fine | Cloudy | Fine |

The quantity of Rain fallen in June, was 2 inches and 20-100ths.

